# Temporary Pacing for Electric Cardiac Stimulation and Neuromodulatory Cardiovascular Therapy

**DOI:** 10.1007/s13239-025-00780-3

**Published:** 2025-04-10

**Authors:** Charles Stark, Pavan Bhat, Eric Rytkin, Igor R. Efimov

**Affiliations:** 1 Department of Biomedical Engineering, Northwestern University, Evanston, IL 60208 USA; 2 Medical Scientist Training Program, Northwestern University Feinberg School of Medicine, Chicago, IL 60611 USA; 3 Department of Medicine (Cardiology), Northwestern University, 303 E Superior St, SQBRC Building, Rm. 11-529, Chicago, IL 60611 USA

**Keywords:** Temporary cardiac pacing, Transvenous temporary pacing, Pacemaker, Arrhythmia, Vagus nerve stimulation, Transcutaneous vagus nerve stimulation

## Abstract

The widespread prevalence and significant consequences of cardiac arrhythmias have been addressed by adopting cardiac stimulation and neuromodulation implantable devices. The oldest, most commonly employed, and most well-known technology is the permanent transvenous cardiac pacemaker. However, in select emergent clinical scenarios and transient pathologies, temporary pacing is preferred. More recently, neuromodulatory vagal nerve stimulation has emerged to address neurologic, psychiatric, and nociceptive pathologies, generating significant clinical and scientific interest in the invention of temporary corollary devices for a subset of indications of nociceptive origin. The dominance of particular implant approaches and anatomic targets in both temporary pacing and neuromodulation in the clinic is owed to capabilities and limitations present in the current technological landscape. However, recent innovations in industry and academia may lead to a fundamental shift in how temporary pacing and neuromodulation are delivered in terms of procedural approach and patient outcomes. In this review, we present an overview of contemporary temporary pacemakers, neuromodulatory therapies, and devices, highlighting novel temporary pacing technologies from the clinic, industry, and academia, such as temporary permanent pacemakers, innovations in non-blood-contacting devices, bioresorbable pacemakers, and advances in neuromodulatory approaches.

## Introduction

The prevalence of cardiac arrhythmias worldwide is estimated to be 1.5–5% [1]. Arrhythmias can have a wide range of effects; while some occur without symptoms, many can lead to sudden cardiac death, which is the leading cause of death in the U.S. [2]. Having been first demonstrated in the 1950s, pacemaker devices have become ubiquitous for the treatment of certain arrhythmias, with over three thousand different models of pacemakers introduced to date in the USA alone [3]. In the U.S., pacemaker indications have been set by a joint group of the American College of Cardiology (ACC), the American Heart Association (AHA), and the Heart Rhythm Society (HRS), with similar guidelines present in the European Society of Cardiology [4]. These ACC/AHA/HRS guidelines recommend permanent pacemaker (PPM) implantation in a tiered classification system for various conditions [5]. For a given condition, a Class I indication represents conditions where the benefits of the PPM device outweigh the risks, a Class II indication represents a divergence of opinion on the efficacy of PPM implantation, and Class III represents contraindications [4]. An estimated three million people worldwide live with a PPM, with the most common use being for a sinoatrial (SA) node dysfunction or a high degree of atrioventricular (AV) block, usually manifesting as bradyarrhythmia or bradycardia [6]. The pacemaker device itself operates with two or three main components: a battery-powered pulse generator (PG), a lead (in most cases), and an electrode, which interfaces with the atrial and/or ventricular surface and provides electric stimulus or electrical sensing. The latest innovations in pacemaker technology have been “leadless” pacemakers, where the battery, PG, and electrode are all on one device.

While PPM implantation has become an effective and robust treatment for many cardiac complications, there are potential acute and chronic deleterious effects of PPMs. In terms of acute complications of traditional, lead-based PPMs, the most common are lead dislodgement (5%), pocket hematoma (3.5%), pneumothorax (1–3%), and thrombosis (1%) [7]. For recently introduced leadless pacemakers, the safety profile has been shown to be significantly better in terms of hematoma, electrode dislodgement, and lead fracture, although safety is highly correlated with operator skill [8]. In some cardiovascular procedures, PPM implantation is optional, such as in the aftermath of transcatheter valve implantation (TAVI); in these cases, some studies have reported that PPM implantation can reduce mortality [9]. However, PPMs introduce the possibility of pacemaker dependency (PD), which is when the heart loses the intrinsic ability to pace itself. The exact electrophysiological mechanism behind pacemaker dependency has not been elucidated, and there is no standard medical test that defines PD; current techniques revolve around setting the pacemaker to a low (30–40 BPM) rate and then assessing whether the heart regains intrinsic function [10]. Due to the incomplete definition of PD, rates have been reported anywhere from 2.1 to 16% post-PPM implantation [11]. In a prospective study of 802 patients with an implanted PPM, Grimm et al. discovered that follow-up duration after PPM implant, presence of atrial fibrillation prior to implant, and presence of second or third-degree AV block at implant, among other clinical variables, were associated with PD [12]. Thus, cardiac pacing can lead to irreversible outcomes, and the decision to implant a PPM must be weighed against the risks of PD.

In contrast to permanent cardiac pacing, temporary cardiac pacing is employed to maintain adequate cardiac hemodynamics in the case of transient cardiac arrhythmias, prophylactically when an acute risk of cardiac arrhythmia is present, or as a bridge therapy prior to PPM implantation. Temporary cardiac pacing is established by using a temporary pacemaker (TPM). It is estimated that over 200,000 TPMs are placed in the US alone per year, and nearly 16,000 of these TPMs are implanted in patients with a complete heart block following an ST-segment elevation myocardial infarction (STEMI), an emergency clinical situation [13]. In an emergency where a patient requires pacing more quickly than a PPM can be placed, a TPM, in one of many forms, should be used in the interim. A TPM can also be used in lieu of a PPM if the etiology of a patient’s arrhythmia is addressable in the short-term, such as an acute STEMI, to mitigate the risk of PD. Prophylactic use of TPMs has also been explored. The incidence of atrial fibrillation after open-heart surgery has been reported to be as high as 40% [14]; prophylactically-implanted epicardial TPMs have been reported to reduce this incidence via biatrial overdrive pacing after certain procedures [15, 16] and is recommended by the American College of Chest Physicians [17].

## Contemporary Temporary Pacing Modalities

### Transcutaneous Pacing

TPMs can be implemented via a transcutaneous, transvenous, epicardial, or transesophageal approach. Transcutaneous pacing boasts the fastest and least invasive setup and is, therefore, the first attempted method of temporary pacing in the emergent setting. However, this approach has a much lower chance of myocardial capture compared to temporary transvenous pacing (TTVP); for this reason, TTVP is the second-line therapy in emergent scenarios. Unfortunately, transcutaneous pacing is uncomfortable for patients due to the high amperage required, which results in muscle stimulation outside of the heart. Minimal transcutaneous pacing output ranges from 5 to 10 milliamperes (mA), while the maximal output may reach 200 mA; the pacing threshold amperage is typically within 40 to 140 mA with a pulse width of 20–30 milliseconds [18]. As delivered energy is the product of pulse width and pacing output, these parameters place transcutaneous pacing’s maximum delivered energy at 6 millijoules, a 400-fold increase compared to transvenous endocardial pacing’s 15 microjoules of delivered energy [19].

### Transvenous Pacing

The predominance of TTVP is due to its high chance of myocardial capture and its superior durability, on the order of weeks, compared to epicardial pacing. In addition, TTVP implantation requires minimal time and causes minimal trauma to the patient. The leads of a TPM can also be repurposed and attached to an implantable PG to form a PPM, obviating the need for lead implantation at a later point. Transvenous implantation is a percutaneous procedure. Briefly, the Seldinger technique is used to advance a pacing lead to the endocardial surface of the right ventricular apex, most commonly via a subclavian vein, the right internal jugular vein, or a femoral vein. Biventricular pacing is indicated in rare cases, and access to the right atrial endocardial surface is acquired. Pacing lead placement is guided by lead markings, which demarcate the extent of lead advancement, continuous electrocardiographic monitoring, and fluoroscopy. Pacing leads are opposed to the endocardial surface with either passive or no fixation [20]. Transthoracic echocardiography may also be used. The procedure should be performed with twilight sedation and local anesthetic [21]. In TTVP, the PG is often extracorporeal, significantly limiting the patient’s mobility and contributing to the risk of infections.

TTVP is considered minimally invasive relative to open-heart surgery as it is a percutaneous procedure. However, while TTVP does not require a thoracotomy, it is blood-contacting, which introduces a new set of potential complications for the recipient. Blood-contacting implantable devices, in particular, pose a risk of sepsis. At the interface of the leads’ electrodes and the site of pacing, thrombosis formation, fibrosis of myocardial tissue, and perforation of the myocardial wall may occur. Damage to the tricuspid valve may result during lead implantation. Acutely, direct trauma can perforate or avulse tricuspid leaflets or disrupt the subvalvular apparatus; subacutely, leaflet impingement, malcoaptation, and regurgitation are possible; chronically, leaflet adhesions, fibrosis, and encapsulations may develop [22]. Finally, calcified thrombo-fibrotic encapsulations may form around the leads, leading to lead-related venous obstruction (LRVO) and venous hypertension. A review of 649,524 Medicare beneficiaries, 65 years and older, receiving a transvenous cardiovascular implantable electronic device between 2015 and 2020 found that 4.3% of patients developed symptomatic LRVO [23]. The median time from implant to LRVO diagnosis was 0.6 years [23]. TTVP, like PPM, is often not possible for neonates, especially premature neonates, due to their smaller body size [24]. TTVP can also limit patients' mobility. Pacing via a subclavian vein limits the comfortable range of motion (ROM) in the affected arm, and patients paced via the femoral vein must remain supine and straight-legged for the duration of pacing. This immobility reduces a patient's quality of life and further increases the risk of deep vein thrombosis (DVT). Thus, femoral vein access is incompatible with longer-term temporary pacing. Femoral access is also associated with a higher risk of lead infection as compared to subclavian access [25]. Finally, because temporary pacing leads passively fix to the endocardial surface, there is a significant risk of dislodgement resulting in intermittent loss of capture. In the case of femoral TTVP leads, the risk of lead dislodgement has been reported to be between 10 and 30% [26]; one study of 100 patients found a 17% risk of dislodgement was found at 4.8 days after lead placement [27].

A conservative choice of temporary rather than permanent pacing may prevent a patient with a transient arrhythmia from developing PD. Still, it should be noted that TPMs can also induce PD. Because TTVP leads are less securely implanted than PPMs, their spontaneous dislodgement may cause a patient, previously not thought to have PD, to experience asystole [28]. The benefit of choosing to implant a TPM must be weighed against the probability of the patient eventually requiring a PPM. PD notwithstanding, the placement of a TPM prior to PPM placement can be hazardous and lead to sepsis. When patients arrive at a hospital that lacks the capability to install a PPM, they may be transferred to a cardiac center with TTVP in situ. Hildick-Smith and Petch [29] have highlighted that this practice can lead to sepsis; Aggarwal et al. [30] found that there is a six-fold increased risk of PPM infection in patients transferred for PPM placement with TTVP in situ. Beyond infection, TTVP carries with it all the risks of central venous access, such as air embolism, while sullying a potential central venous access site. For these reasons, Hildick-Smith and Petch strongly advise against the use of TTVP for patient transport unless absolutely necessary. Nevertheless, bridge pacing is advantageous in certain instances. In an observational study of 688 patients, Chang et al. [31] found that a longer one-month temporary-permanent pacemaker bridge following transcatheter aortic valve replacement, used to determine if the patient’s conduction system has been temporarily or permanently altered, was superior to a two-day bridge and resulted in a significant decrease in eventual PPM placement. Thus, the decision on whether to implant a TTVP and how long to maintain pacing must be made on a procedure-by-procedure basis, weighing the risks of PD and TTVP complications accordingly.

### Epicardial Pacing

Epicardial pacing, conversely, is non-blood-contacting, lowering the risk of infection and thromboembolism as compared to TTVP. There is also a lower risk of heart valve damage and perforation of the heart. Additionally, epicardial pacing is preferable for young infants and neonates over TTVP due to their smaller body size [32]. Yet, currently, this approach is used exclusively following open-heart surgery to prophylactically address postoperative arrhythmias because epicardial pacing lead implantation requires access to the pericardial space. Four days postoperatively, atrial and ventricular pacing thresholds begin to rise for epicardial TPMs, resulting in failure to capture myocardium [33]. Most importantly, this approach currently requires a second invasive surgery to retrieve the TPM.

### Transesophageal Pacing

The least commonly employed approach is transesophageal pacing. Briefly, a pacing or recording catheter is introduced via the nasal canal or mouth and used to either pace the atria, terminate atrial fibrillation or flutter, or record atrial electrical waveforms for diagnostic purposes. This approach is limited to atrial tissue, has a poor chance of myocardial capture compared to TTVP, and is uncomfortable for patients. It is therefore not often used.

## Neuromodulation

While the most axiomatic reason for a recurrent, stimulating response is to deliver a pacing stimulus of the cardiac muscle, there are many other cases where small, imperceptible shocks contain clinical value in cardiology and beyond.

### Low-Level Vagal Nerve Stimulation for Cardiac Applications

One such example is low-level vagal nerve stimulation (LLVNS) for the modulation of arrhythmias and inflammation [34]. The vagus nerve is a long nerve that runs from the base of the brain to the stomach and is involved in controlling many autonomic functions [35]. FDA-approved therapies already exist for the treatment of epilepsy and depression using VNS [36]. LLVNS for cardiac applications is a rapidly developing field and many pilot studies have shown that temporary VNS can inhibit ventricular arrhythmogenesis in animal models [33]. Similarly, clinical pilot studies have demonstrated that temporary vagus nerve stimulation after cardiac surgery can reduce postoperative inflammation [37]. Specifically, in myocardial ischemia, VNS during reperfusion has been shown to reduce infarct size [38].

### Mechanisms and Applications of VNS

While the mechanisms are not precisely known, several key signaling pathways have been implicated, mainly nitric oxide (NO), the GIRK channel, and parasympathetic nervous system activation. It is also possible to stimulate the vagus nerve to become pro-arrhythmic by modulating the stimulation protocol, which can be useful for research purposes [14]. Another research application of this kind of stimulation is the controlled induction of asystole, which can be useful in surgery when short-term asystole is desired [38].

### Clinical Use of VNS in Heart Failure

In addition to ischemia and arrhythmia, VNS has also been used in clinical trials for congestive heart failure (CHF). The overarching hypothesis is that a mismatch of sympathetic and parasympathetic activation can lead to CHF, and three large clinical studies showed that VNS improves multiple quality-of-life markers in CHF patients [39]. Invasive VNS devices operate under the same principles as a pacemaker, with a pulse generator, leads, and electrodes, usually implanted with the electrodes contacting the vagus nerve within the neck and the PG “can” in the subclavian space.

## Summary and Limitations of Current Methods

### Temporary Pacing

In summary, there are clear advantages and disadvantages to each temporary pacing method. Transcutaneous pacing is quick to set up but uncomfortable for the patient and often fails to capture. TTVP boasts a better capture likelihood with reasonable setup speed and is a percutaneous procedure, but its blood-contacting nature introduces significant risks for complications, despite commonly being deemed “minimally invasive”. Epicardial pacing also has improved capture likelihood and does not share the risks of TTVP inherent in blood-contacting approaches. However, epicardial pacing is only an option following thoracotomy and requires a second surgery to retrieve the pacemaker while also introducing the risk of pericardial adhesions. Clearly, there is a need in the emergent, transient arrhythmia, and arrhythmia prophylaxis spaces to improve temporary pacing technologies. In the emergent setting, there is a need for a TPM that has both the setup speed of a transcutaneous approach and the enhanced capture rate of a transvenous approach, which does not require a cardiac center. To address transient arrhythmias more safely, there is a need for a solution that does not require thoracotomy and does not significantly increase the likelihood of sepsis, myocardial perforation, tricuspid valve damage, thromboembolism, or LRVO, either by not contacting blood or by other means. Post-procedural arrhythmia prophylaxis measures that do not require an additional procedure for device explant are also needed.

Both transvenous and epicardial TPMs require a follow-up removal procedure when temporary pacing is no longer required. This removal introduces additional risk to patients. TPMs with fully implanted power sources require an explant to cease pacing. This time between a clinician recognizing that temporary pacing is no longer required and the explant procedure represents a window in which PD can progress. Unfortunately, the alternative to implanted PGs are extracorporeal TPM power sources. These extracorporeal PGs significantly limit patient mobility, reducing patient quality of life and increasing the risk of DVTs. Finally, as Sunwoo et al. [40] point out, inflexible implantable cardiac devices disrupt cardiac hemodynamics. TPMs—whose leads are often stiffer than PPMs—may, therefore, disrupt cardiac hemodynamics to a greater extent than PPMs, representing a need for TPMs which more closely match the stiffness of native cardiac tissue.

### Temporary Neuromodulation

In addition to temporary pacing, neuromodulatory therapies such as vagal nerve stimulation (VNS) present both promising benefits and significant limitations in the management of cardiovascular conditions. Temporary neuromodulation can serve as an alternative to traditional temporary pacing for the treatment of postoperative cardiac abnormalities. However, contemporary neuromodulation methods exhibit several disadvantages. Traditional cervical neuromodulation, for instance, is invasive and requires a surgical procedure for implantation, necessitating different surgical specialties compared to temporary pacemakers that directly contact the heart. This complexity can be prohibitive in the context of postoperative temporary pacing following cardiac procedures. Despite these challenges, neuromodulation holds the highest potential for treating postoperative atrial fibrillation [41].

Another critical limitation is that neuromodulatory treatments are still undergoing extensive testing. The long-term efficacy and safety of temporary VNS have not been fully established. Challenges include maintaining device stability, achieving precise nerve fiber targeting, and requiring frequent adjustments to stimulation parameters to optimize therapeutic outcomes. While LLVNS addresses the invasiveness of traditional neuromodulatory approaches by being non-invasive, it has yet to demonstrate the same level of efficacy as direct surgical neuromodulation that contacts the nerves themselves. Additionally, non-invasive LLVNS methods may suffer from reduced precision in stimulation, potentially leading to suboptimal therapeutic effects and variability in patient responses. The lack of direct nerve contact can also result in weaker signal transmission, limiting the overall effectiveness of the treatment. Furthermore, non-invasive approaches may require more frequent sessions to achieve desired outcomes, increasing the burden on both patients and healthcare providers. These disadvantages highlight the need for further advancements in temporary neuromodulation technologies to enhance their efficacy, safety, and practicality in clinical settings. Addressing these limitations through advancements in device design, such as the development of non-blood-contacting and bioresorbable neuromodulatory devices, as well as refining stimulation algorithms and enhancing regulatory pathways, is essential for improving the safety, efficacy, and applicability of temporary neuromodulation in cardiovascular therapy.

## Innovations in Temporary Pacing

### Substernal Pacing

To address the aforementioned unmet needs, novel pacing technologies have been developed by medical device ventures and research groups. AtaCor Medical, Inc., founded in 2014, has developed an extracardiac, substernal pacing lead deployment system that places pacing leads in the anterior mediastinum. Presently, AtaCor’s EV-ICD Lead System and EV Temporary Pacing System for temporary pacing are undergoing clinical trials for use in emergency cardiac defibrillation and temporary pacing, respectively. The feasibility of such a substernal pacing approach, from both implantation and myocardial capture perspectives, was supported by Quast et al. [42], and the first successful implantation of and defibrillation with AtaCor’s EV-ICD Lead System was reviewed by Burke et al. [43]. Bressi et al. [44] comment further on this first-in-human implant’s compatibility with DF-4 standardized connectors for implantable cardiac defibrillator’s PGs. Given the promise of substernal pacing for defibrillation and temporary pacing, Medtronic Inc. has also entered the substernal defibrillation space with its Aurora EV-ICD system, also supported by a feasibility study conducted by Brouwer et al. [45]. While the 2017 ASD clinical study confirmed the efficacy of substernal cardiac defibrillation [46], the 2018 SPACE clinical study demonstrated the efficacy of substernal temporary pacing and suggested the possibility of simultaneous substernal electrocardiography in future devices [47]. The 2019 multicenter ASD2 study, conducted in 79 patients, demonstrated the ability of substernal leads to pace, defibrillate, and sense the heart’s conduction system with a median implantation time of 12 ± 9 minutes [48]. Thus, the substernal approach shows tremendous promise as a non-blood-contacting temporary pacing solution to address the needs presented by TTVP in the emergent and transient arrhythmia settings.

### Temporary Permanent Transvenous Pacemakers

Existing technology has also been leveraged to improve clinical outcomes in temporary pacing. Given the risks of infection and lead dislodgement presented by TTVP, temporary permanent pacemakers (TPPM) with actively fixed endocardial surface leads have been employed. This technique entails anchoring the electrode by penetrating the endocardial surface, most commonly via a corkscrew-shaped mechanism. TPPM placement is similar to transvenous PPM placement, except the PG is extracorporeal and taped to the body. Therefore, a subcutaneous prepectoral pocket for the PG is not needed. Active fixation transvenous pacing (ATVP) is thought to decrease the probability of lead dislodgement and makes more endocardial sites possible for pacing. However, ATVP is considered more invasive than passive fixation of leads and requires fluoroscopic guidance [49]. A 2019 review of 24 studies representing 770 patients by Suarez and Banchs recommends TPPM use with ATVP as a first-line therapy over passive fixation TTVP when patients are stable enough to be transferred to a room with fluoroscopy, especially for patients who may require pacing for longer periods of time. [50]. This review found a 1.7% risk of dislodgement for TPPMs, which is a 90% relative risk reduction or a 15.3% absolute risk reduction as compared to the 17% risk of dislodgement for passively fixed TTVP. However, this 17% risk was found for femoral TTVP alone, a technique that is no longer favored. As a result of ATVP’s lower probability of lead dislodgement, Suarez and Banchs report a lower risk of lead infection. In addition, the cost of care for patients who require temporary pacing for greater than 18 hours is diminished when ATVP is used instead of passive fixation, chiefly due to the ability of TPPM patients to ambulate and in some instances be discharged from the hospital with a TPPM [51].

### Bioresorbable Epicardial Pacing

Novel technological developments from academic laboratories are poised to address the remaining needs in temporary pacing. The fully implantable, bioresorbable, flexible, leadless, and wirelessly powered TPM reported by Choi et al. [52] directly addresses the major drawbacks of contemporary TPMs. Once temporary pacing is no longer indicated, a second procedure is needed to explant the contemporary TPM; until then, patients must remain wired to an external power source. Bioresorbability obviates the need for a follow-up explant procedure, and wireless pacing both untethers the patient from an external power source and allows for instantaneous cessation of cardiac pacing once no longer indicated, combining the greatest strengths of implantable and extracorporeally powered TPMs alike. The device is non-blood-contacting as it is leadless, avoiding the complications presented by TTVP previously mentioned, and its flexible nature minimizes its impact on cardiac hemodynamics and wall motion. Similar devices are small enough to be implanted in rodent models [53] and are fully transparent [54], facilitating TPM research in small animal models and with optical mapping analysis, respectively. To address the temporary pacing need presented by acute STEMI, Ryu et al. [55] modified the device to provide localized mechanical support and alternate conductive pathways for ischemic cardiac tissue.

### Percutaneous Epicardial Pacing

Challenges remain for neonates in need of temporary pacing as epicardial pacing requires a thoracotomy while TTVP is contraindicated due to small patient body size. Neonates may require temporary pacing due to hemodynamic instability secondary to bradycardia or congenital heart block. To address this care gap, Kumthekar et al. 2023 reported their development of an epicardial lead placed via a minimally invasive, percutaneous approach with an extracorporeal PG [56]. The feasibility of this approach for neonates was demonstrated by successfully performing lead implants in piglets of comparable weight to neonates. In brief, a trocar modified to provide intrathoracic illumination houses a Veress needle for intrathoracic deployment. Pericardial access is obtained by a dilator modified to allow for direct visualization of sheath insertion via a fiber optic camera. A splitter tool at the proximal end of the deployment device allows both fiberscope and pacing leads to advance to the pericardial space. All six piglets received percutaneous epicardial pacing with pacing thresholds at 1 millisecond pulse-widths between 0.7 and 2.8 millivolts without procedural complications. The median total procedure time was reported to be less than 20 minutes. This delivery method has significant advantages: it is both a percutaneous and non-blood-contacting procedure; it is small enough to be used for neonates; and it can be directed to multiple sites for epicardial pacing, including the atria. However, the authors point out that there are currently no active fixation pacing leads designed for an approach tangential to the epicardial surface that are strong enough to accommodate the somatic growth of the patient [57]. Still, a percutaneous epicardial pacing method, if shown to be safe in humans, could lead to a pacing option that accommodates the needs of neonates. Further study in humans is necessary.

## Innovations in Neuromodulation

Current VNS devices are implanted invasively and, therefore, pose risks to the patient, limiting their use as a neuromodulatory cardiovascular therapy.

### Transcutaneous VNS Approaches

Recently, transcutaneous VNS has been explored via the outer acoustic ear canal and proposed as an alternative to the invasive approach. The TREAT-AF study, a prospective double-blind, sham-controlled, randomized clinical trial examining the effect of chronic low-level tragus stimulation (LLTS) in patients with paroxysmal AF, found that chronic, intermittent LLTS resulted in lower AF burden as assessed by noninvasive continuous electrocardiogram monitoring at baseline, 3 months, and 6 months, heart-rate variability, and tumor necrosis factor-alpha serum levels [58]; this study supported the use of LLTS as a treatment for paroxysmal AF in select patients. Although transcutaneous VNS is easily accessible, it requires external electrodes on the ear lobe, which need to be constantly attached to a battery pack [36].

### Emerging Transient and Bioresorbable VNS Devices

A small, temporary vagus nerve stimulation device can be conceived to be used immediately following a cardiac procedure to improve outcomes associated with post-operative trauma. Such a transient, bioresorbable device has recently been developed by Lee et al, which uses ultrasound to stimulate triboelectricity to both generate the power to stimulate the sciatic nerve and resorb the device [59]. While these transient electronics are being developed, the feasibility of temporary neuromodulation using traditional pulse-generating and cuff-electrode devices for splenic nerve stimulation following esophagectomy has advanced to a clinical trial [60]. Similarly, such a form of device could be used to stimulate the vagus nerve for transient cardiac modification following surgical procedures.

## Conclusions

**Fig. 1:**
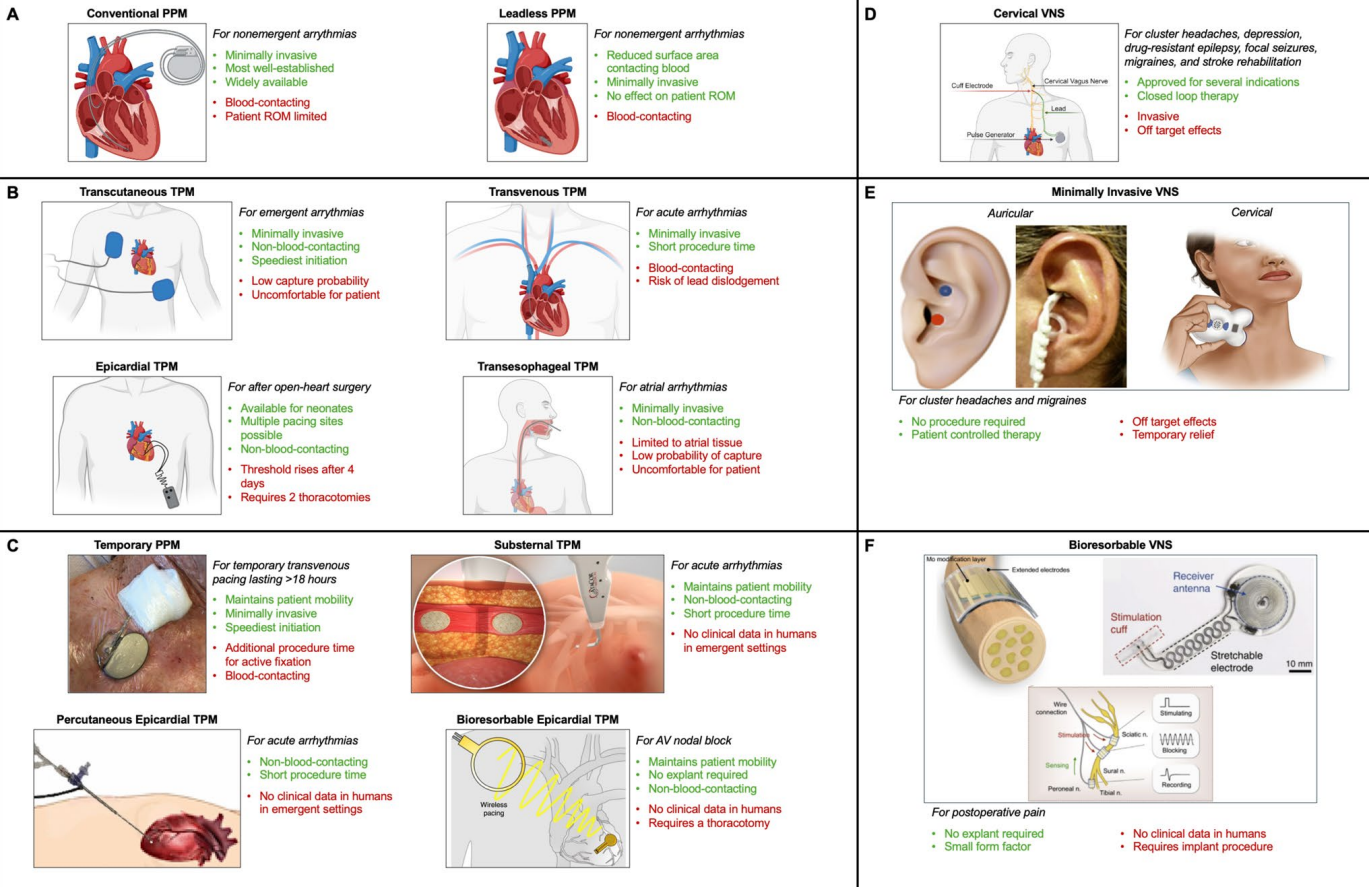
Overview of permanent and temporary pacemakers and neuromodulators. **a** Permanent pacemakers. **b** Contemporary temporary pacing methods. **c** Innovations in temporary pacing from industry and academia. Italics denote indications. Green bulleted text denotes advantages. Red bulleted text denotes disadvantages. Icons are borrowed and modified from: **a** Conventional and Leadless PPM [60]; **b** Transcutaneous TPM, Transvenous TPM, Epicardial TPM, Transesophageal TPM [61]; **c** Temporary PPM [50], Substernal TPM [62], Percutaneous Epicardial TPM [63], Bioresorbable Epicardial TPM [52], **d** Cervical VNS [61]; **e** Minimally Invasive VNS [64, 65], **f** Bioresorbable VNS/Neuromodulation [66–68]

TPMs can be used in lieu of PPMs for transient arrhythmias and to prophylactically manage post-procedural arrhythmias. They are a crucial therapy in emergent settings such as acute STEMI. At present, the predominant TPM implantation approach is TTVP, but the limitations inherent in this approach, combined with recent innovations in the medical device industry and academia, suggest that previously less viable approaches like epicardial pacing may see greater usage in the future. The TTVP approach introduces potentially fatal risks such as sepsis and thromboembolism as it is blood-contacting. There is a significant need for TPMs that are non-blood-contacting, quickly implantable, do not require a second procedure for explant, and are feasible for use in vulnerable pediatric patients like neonates. In addition, the risk of PD, DVT, and suboptimal cardiac hemodynamics necessitate TPMs with instantaneously togglable pacing, no limits on patient mobility, and device compliance comparable to cardiac tissue, respectively. As with temporary pacing, there is a similar need to reduce the risk to patients undergoing VNS and adopt minimally invasive techniques like LLTS. The innovative substernal pacing approach currently being developed by the medical device industry and novel, soft, bioresorbable implantable devices created by academic researchers represent paradigm shifts in temporary cardiac pacing and neuromodulatory cardiovascular therapy, which are posed to address these crucial needs. Table 1 and Fig. 1 summarize the indications, advantages, and limitations of both conventional and emerging temporary pacing devices for electric cardiac stimulation and neuromodulatory cardiovascular therapy.

**Table 1:** Overview of permanent and temporary pacemakers and neuromodulators

Device name	Description	Indication(s)	Advantages	Limitations
Conventional PPM	Pacing leads are advanced to the heart through the venous system via the Seldinger technique and pacing electrodes are actively fixed to the endocardial surface of one or more sites; the PG is typically implanted in the subcutaneous, prepectoral space contralateral to the patient’s dominant hand	Bradyarrhythmias, tachyarrhythmias	Minimally invasive, most well-established, widely available	Blood-contacting, PG limits patient ROM
Leadless PPM	Pacing electrodes are advanced to the heart through the venous system via the Seldinger technique and actively fixed to the endocardial surface of one or more sites; the pulse generator is low-profile and remains in the chamber of the heart, directly attached to the electrodes without leads	Bradyarrhythmias, tachyarrhythmias	Decreased surface area in contact with blood	Blood-contacting
Transcutaneous TPM	Adhesive electrodes are placed on the anterior and left-lateral chest; the PG is an extracorporeal pacing box	Acute hemodynamic-compromising arrhythmias	Minimally invasive, speediest initiation	Low capture probability, patient discomfort
TTVP	Pacing leads are advanced to the heart through the venous system via the Seldinger technique and passively fixed to the endocardial surface of one or more sites; the PG is an extracorporeal pacing box	Acute hemodynamic-compromising arrhythmias	Minimally invasive, relatively speedy procedure	Blood-contacting, risk of lead dislodgement
Epicardial TPM	After open heart surgery, pacing leads are advanced to the pericardial space and actively fixed to one or more potential sites on the epicardial surface including atria and ventricles; the PG is an extracorporeal pacing box	Arrhythmia prophylaxis, pediatric open heart surgery patients, postprocedural bradycardia	Available for neonates, multiple pacing sites possible, non-blood-contacting	Pacing threshold increases four-days post-operatively, requires an additional thoracotomy for explant
Transesophageal TPM	A pacing lead are advanced to the esophagus at the level of the posterior atria under anesthesia via the nasopharynx or oropharynx; the PG is an extracorporeal pacing box	Atrial waveform measurement, AF, Atrial Flutter	Minimally invasive, non-blood-contacting,	Limited to atrial tissue, low capture probability, patient discomfort
TPPM	Pacing leads are advanced to the heart through the venous system via the Seldinger technique and pacing electrodes are actively fixed to the endocardial surface of one or more sites; the PG remains extracorporeal, typically adhered to the patient’s chest	Bradyarrhythmias, tachyarrhythmias	Lowered chance of lead displacement, minimally invasive	Additional procedure time for active fixation, blood-contacting
Substernal TPM	A pacing lead is advanced to the anterior mediastinum via a subxiphoid approach; the PG is implanted in a subcutaneous pocket in the left midaxillary space, and the proximal lead is tunneled to the PG	Acute hemodynamic-compromising arrhythmias	Minimally invasive, non-blood-contacting, short procedure time	No clinical feasibility data available for use in emergent scenarios
Percutaneous epicardial pacing	A pacing lead is introduced to the pericardial space via percutaneous injection and is actively fixed to the epicardial surface; the PG remains extracorporeal	AV block in neonates, bradycardia in neonates	Available for neonates, minimally invasive, multiple pacing sites possible, non-blood-contacting, percutaneous, short procedure time	No clinical feasibility data available in humans
Bioresorbable Epicardial TPM	The device is advanced to the pericardial space by pericardiotomy and adhered directly to the epicardial surface; power is delivered wireless by an extracorporeal power source	Atrioventricular nodal block	Lower-profile device than conventional epicardial TPMs, no additional thoracotomy for explant required	No clinical feasibility data available in humans, requires thoracotomy for implant
Cervical VNS	A small incision is made at the neck, and the left vagus nerve is dissected away from surrounding tissue, and a subcutaneous pocket is created inferior to the left clavicle. A cuff electrode is threaded and wrapped around the vagus nerve and connected to the pulse generator.	Drug-resistant epilepsy, depression, focal seizures, stroke rehabilitation, cluster headaches and migraines	Can replace or supplement chronic medication	Battery life is typically 3–5 years, procedure can cause initial discomfort due to off-target effects.
Non-invasive VNS	The vagus nerve is stimulated epidermally, either from the auricular branch or the cervical branch, beneath the carotid sheath. A pulse generating device is either held at the neck or clipped at the ear and generates an electric field sufficient to stimulate the underlying vagus nerve.	Cluster headaches and migraines	Patient has greater control on when to administer therapy, no surgery required	Can have off-target effects, requires patient compliance
Bioresorbable VNS	A small, bioresorbable device is implanted around the carotid nerve, delivering programmed stimulation, and slowly biodegrades over time. Can be self-powered or powered via an external stimulus.	Post-operative pain	No retrieval procedure for device, implantation procedure is simpler	Less control possible due to small form factor, may require external powering device
